# Molecular Lymph Node Staging with One-Step Nucleic Acid Amplification and its Prognostic Value for Patients with Colon Cancer: The First Follow-up Study

**DOI:** 10.1007/s00268-020-05949-6

**Published:** 2021-01-29

**Authors:** Benjamin Weixler, Sofia Teixeira da Cunha, René Warschkow, Nicolas Demartines, Ulrich Güller, Andreas Zettl, Alexander Vahrmeijer, Cornelis J. H. van de Velde, Carsten T. Viehl, Markus Zuber

**Affiliations:** 1 Department of Surgery, Leiden University Medical Centre, Leiden, The Netherlands; 2 Department of General, Visceral and Vascular Surgery, Charité University Hospital, Campus Benjamin Franklin, Berlin, Germany; 3 Department of Surgery, Cantonal Hospital Olten, Olten, Switzerland; 4 Department of Surgery, Cantonal Hospital St. Gallen, St. Gallen, Switzerland; 5 Department of Surgery, Vaudois University Hospital Centre, Lausanne, Switzerland; 6 Department of Oncology, Spital STS AG, Thun, Switzerland; 7 Department of Pathology, Viollier AG, Basel, Switzerland; 8 Department of Surgery, Hospital Centre Biel, Biel, Switzerland; 9 Visceral Surgery Centre Clarunis, St. Clara Hospital and University Hospital Basel, University of Basel, Spitalstrasse 21, CH-4031 Basel, Switzerland

## Abstract

**Background:**

Molecular lymph node workup with one-step nucleic acid amplification (OSNA) is a validated diagnostic adjunct in breast cancer and also appealing for colon cancer (CC) staging. This study, for the first time, evaluates the prognostic value of OSNA in CC.

**Patients and methods:**

The retrospective study includes patients with stage I-III CC from three centres. Lymph nodes were investigated with haematoxylin and eosin (H&E) and with OSNA, applying a 250 copies/μL threshold of CK19 mRNA. Diagnostic value of H&E and OSNA was assessed by survival analysis, sensitivity, specificity and time-dependent receiver operating characteristic curves.

**Results:**

Eighty-seven patients were included [mean follow-up 53.4 months (± 24.9)]. Disease recurrence occurred in 16.1% after 19.8 months (± 12.3). Staging with H&E independently predicted worse cancer-specific survival in multivariate analysis (HR = 10.77, 95% CI 1.07–108.7, *p* = 0.019) but not OSNA (HR = 3.08, 95% CI 0.26–36.07, *p* = 0.197). With cancer-specific death or recurrence as gold standard, H&E sensitivity was 46.7% (95% CI 21.3–73.4%) and specificity 84.7% (95% CI 74.3–92.1%). OSNA sensitivity and specificity were 60.0% (95% CI 32.3–83.7%) and 75.0% (95% CI 63.4–84.5%), respectively.

**Conclusions:**

In patients with CC, OSNA does not add relevant prognostic value to conventional H&E contrasting findings in other cancers. Further studies should assess lower thresholds for OSNA (< 250 copies/μL).

## Introduction

In the last decades, improvements in surgical techniques as well as in pathological assessment and adjuvant therapy could significantly enhance 5-year survival rates of patients with colon cancer. Positive lymph node (LN) status (stage III) is still one of the most important prognostic factors determining the allocation of adjuvant chemotherapy [1]. Most interestingly, there exists no real gold standard for reliable identification of negative and positive LN [2]. The standard histopathological LN workup consists of a haematoxylin and eosin (H&E) staining of one to two sections of each retrieved LN. Such conventional histopathological workup carries the substantial risk of “understaging” due to undetected micro- or macrometastases (i.e. tissue allocation bias). A minimum of 12 analysed LN is therefore recommended by most national cancer organizations [3, 4]. However, the agreement to yield a minimum of 12 LN was proposed over two decades ago and up to date clear evidence to justify this threshold is still missing [5, 6]. Furthermore, determination of a certain number of LN cannot compensate for a missing “gold standard” and the inherent tissue allocation bias. Understaging may therefore substantially contribute to the high recurrence rate (20–25%) of patients with negative LN status [7]. It was demonstrated that multilevel LN sectioning combined with immunohistochemistry can improve the detection rate of small nodal tumour infiltrates (i.e. isolated tumour cells and micrometastases), though it is a costly and protracted process [8]. Considering the possible tissue allocation bias as well as the cost and time intensive multilevel sectioning, molecular methods like one-step nucleic acid amplification (OSNA) are appealing since they do not require microscopic examination, and are standardized and fast. For OSNA, cytokeratin 19 (CK19) mRNA is amplified based on reverse transcription-loop-mediated isothermal amplification [9]. CK19 is one of the most widely used immunohistochemical marker to identify epithelial cells and is highly suggestive for the presence of colon cancer metastases when detected in a respective LN [10–12].

Up to date, only a few studies evaluated the clinical performance of OSNA in colon cancer. These studies compared OSNA with conventional pathological workup and immunohistochemistry techniques showing a high concordance level between the investigated methods [12–16]. However, the prognostic value of OSNA and thus its suitability as a “gold standard” to distinguish between LN positive and LN negative patients with colon cancer is unknown as respective data are entirely missing. The purpose of the here presented study was to evaluate the prognostic value of OSNA in patients with stage I-III colon cancer.

## Patients and methods

For this retrospective study, patients with Union for International Cancer Control stage I, II and III colon cancer were identified from three centres—Cantonal Hospital Olten, Switzerland, Lausanne University Hospital, Switzerland, and Leiden University Medical Centre, Netherlands—that previously participated in three published cohort studies [13–15], except the patients recruited at the Lausanne University Hospital. Medical ethical approval was obtained from the local ethical committees, and the obtainment of patient consent is mentioned both in detail in the three previous publications [13–15]. The study was performed in accordance with the Declaration of Helsinki.

Included patients were over 18 years old with histological proven colon carcinoma, localized above the peritoneal fold, and submitted to in vivo (Olten [13], Lausanne) or submitted to ex vivo (Olten [14], Leiden [15]) sentinel LN mapping.

Recruitment occurred between January 2007 and December 2013. Follow-up data were retrieved from the patients’ medical files and treating general practitioners until October 2017.

Exclusion criteria were carcinoma in situ, stage IV colon cancer or preoperative neoadjuvant treatment.

The study protocols of the previous cohort studies were in detail mentioned elsewhere [13–15]. In brief, sentinel LN mapping with isosulfan blue (Cantonal Hospital Olten and Lausanne University Hospital) or indocyanine green (Leiden University Medical Centre) was performed during standard oncological colon resection. Forty (22 [13] and 18 [14]) patients from Cantonal Hospital Olten, 21 from Lausanne University Hospital and 26 from Leiden University Medical Centre [15] were included. All harvested LN over 3 mm in greatest dimension [13] or a short axis ≥ 10 mm [15] was cut into 4 slices labelled from a to d. Blocks a and c were stored for later OSNA analysis. Blocks b and d were fixed in neutral buffered formaldehyde, processed in paraffin blocks and allocated to conventional standard H&E staining, multilevel H&E staining and immunohistochemistry (IHC) for CK19 as described elsewhere [13, 15].

In 18 of 40 patients from Cantonal Hospital Olten—the second study [14] – the harvested LN was processed cutting a middle section of 1 mm dedicated to standard H&E. The remaining parts of the LN were investigated by OSNA. Since the main focus of the present study was to evaluate the prognostic value of OSNA analysis, all patients from the three centres were included, despite the slightly different LN processing protocol.

For OSNA analysis, a CK19 mRNA copy threshold of 250 copies/μL was used. A CK19 mRNA copy number of < 250 copies/μL was considered negative for metastatic LN involvement, and a copy number ≥ 250 copies /μL was considered positive.

This was defined by previous studies using the logarithmic midpoint between the maximum value of the CK19 mRNA copy number in LN from pN0 patients and minus 2 standard deviations (SD) [12] or minus 3 SD [17] value from the average of CK19 mRNA copy number in histopathologically positive LN.

These studies also defined the micrometastases threshold between 250 and 4999 CK 19 mRNA copies/ μL. LN with 5000 or more mRNA copies/ μL was considered macrometastases. [12, 17].

As currently no “gold standard” for the definition of “real” negative and positive LN exists, we used the event of cancer-related death and cancer-related recurrence as proof of real node positivity and patients without disease recurrence or cancer-related death during follow-up were indexed to real negative LN.

The 30-day mortality and overall mortality were documented as well as the cause of death. Tumour recurrences were defined by radiological, endoscopic or histological evidence.

### Statistical analysis

Statistical analysis was performed using the R environment (http://www.rproject.org). Two-sided *p* values < 0.05 were considered statistically significant. Continuous data were expressed as the mean ± standard deviation (SD). Confidence intervals (CIs) of binominal proportions were estimated as exact binomial 95% CI. After descriptive analysis of patient and tumour data for centre effects, the impact of detection of metastatic LN by H&E and OSNA on cancer specific survival (CSS), cancer-specific and recurrence-free survival (CSRFS) and disease-free survival (DFS) was assessed by Cox regression analysis with and without risk adjustment for patient and tumour data. Additionally, backward variable selection procedures from the full Cox regression model based on the Akaike's information criterion (AIC) were performed. The proportional hazard assumption was tested by scaled Schoenfeld residuals and by inspection of the hazard ratio (HR) plots. CI for hazard ratios (HR) were estimated with the Wald method. Significance tests were performed as likelihood ratio tests [18]. Then, sensitivity, specificity and diagnostic odds ratio for detection of metastatic LN by H&E and OSNA based on cancer-specific deaths or recurrence as the gold standard for true LN positivity were estimated. To further assess the diagnostic accuracy of H&E and OSNA and the potential influence of their cut-offs (≥ 250 copies /μL for OSNA and one positive LN for H&E), time-dependent receiver operating characteristic (ROC) curves were estimated from the censored survival data [19]. The area under the ROC curve (AUC) was computed using the nonparametric trapezoidal method [20]. Cut-off values were estimated by maximizing the Youden index.

## Results

### Patient and tumour characteristics

A total of 87 patients with stage I-III colon cancer were included in this retrospective study. Patient and tumour characteristics did not differ relevantly between the three centres (Table 1).

**Table 1:** Patient and tumour characteristics comparing the three centres

Characteristic	Total (*n* = 87)		CHO (*n* = 40)		LUH (*n* = 21)		LUMC (*n* = 26)		*p*
	*n*	%	*n*	%	*n*	%	*n*	%	
Gender									
Female	38	43.7	20	50.0	8	38.1	10	38.5	0.548^A^
Male	49	56.3	20	50.0	13	61.9	16	61.5	
Age									
Mean ± SD	71.1 ± 11.7		73.4 ± 10.3		71.4 ± 12.3		67.2 ± 12.7		0.168^A^
Range	38.5–94.4		47.5–92.8		48.1–94.4		38.5–87.8		
Age group									
< 60	13	14.9	4	10.0	3	14.3	6	23.1	0.78 ^B^
60–69	25	28.7	10	25.0	7	33.3	8	30.8	
70–79	27	31.0	14	35.0	6	28.6	7	26.9	
> 79	22	25.3	12	30.0	5	23.8	5	19.2	
Tumour localization									
Ascending colon	40	46.0	14	35.0	11	52.4	15	57.7	0.483^C^
Transverse colon	9	10.3	5	12.5	2	9.5	2	7.7	
Descending colon	10	11.5	4	10.0	2	9.5	4	15.4	
Sigmoid colon	28	32.2	17	42.5	6	28.6	5	19.2	
Staging UICC H&E									
I	24	27.6	13	32.5	4	19.0	7	26.9	0.592^A^
II	39	44.8	19	47.5	10	47.6	10	38.5	
III	24	27.6	8	20.0	7	33.3	9	34.6	
Staging UICC OSNA									
I	21	24.1	11	27.5	3	14.3	7	26.9	0.576^A^
II	38	43.7	16	40.0	9	42.9	13	50.0	
III	28	32.2	13	32.5	9	42.9	6	23.1	
T-stage									
T1	7	8.0	3	7.5	2	9.5	2	7.7	0.636^A^
T2	20	23.0	11	27.5	3	14.3	6	23.1	
T3	52	59.8	23	57.5	12	57.1	17	65.4	
T4	8	9.2	3	7.5	4	19.0	1	3.6	
Lymphatic invasion									
L0	71	81.6	37	92.5	10	47.6	24	92.3	< 0.001^A^
L1	8	9.2	3	7.5	3	14.3	2	7.7	
Lx	8	9.2	0	0.0	8	38.1	0	0.0	
Vascular invasion									
V0	71	81.6	35	87.5	12	57.1	24	92.3	< 0.001^A^
V1	10	11.5	5	12.5	3	14.3	2	7.7	
Vx	6	6.9	0	0.0	6	28.6	0	0.0	
Histological grade									
Well (G1)	7	8.0	1	2.5	4	19.0	2	7.7	0.097^A^
Moderate (G2)	63	72.4	30	75.0	14	66.7	19	73.1	
Poor (G3)	13	14.9	8	20.0	3	14.3	2	7.7	
No information	4	4.6	1	2.5	0	0.0	3	11.5	
Number of retrieved LN for routine H&E									
Mean ± SD	23 ± 9.1		26 ± 9.3		20 ± 9		20.8 ± 7.5		0.013^B^
Range	9.0–60.0		11.0–60.0		9.0–40.0		10.0–33.0		
< 12	7	8	1	2.5	2	9.5	4	15.4	0.162^A^
≥ 12	80	92	39	97.5	19	90.5	22	84.6	
Range	0.0–11.0		0.0–11.0		0.0–9.0		0.0–10.0		
Number of retrieved LN for OSNA									
Mean ± SD	7.5 ± 6.6		12.0 ± 5.8		6.4 ± 5.3		1.3 ± 0.5		< 0.001^A^
Range	1.0–27.0		3.0–27.0		1.0–22.0		1.0–3.0		
Adjuvant therapy									
Yes	14	16.1	5	12.5	5	23.8	4	15.4	0.563^B^
No	73	83.9	35	87.5	16	76.3	22	84.6	
Follow up (months)									
Mean ± SD	53.4 ± 24.9		55.5 ± 23.6		64.0 ± 29.3		41.7 ± 18.2		< 0.001^B^
Range	0.1–109.5		0.8–97.8		4.2–109.5		0.1–64.4		
Recurrence									
Recurrence	14	16.1	6	15.0	4	19.0	4	15.4	0.931^A^
No Recurrence	73	83.9	34	85.0	17	81.0	22	84.6	
Time to recurrence (months)									
Mean ± SD	19.8 ± 12.3		28 ± 13.5		17.8 ± 10.5		10.5 ± 3.0		0.130^C^
Range	2.1–42.0		10.7–42.02		1–30.2		7.4–13.5		
Recurrence localization									
Local	1	1.1	1	2.5	0	0.0	0	0	0.965^A^
Distant	13	14.9	5	12.5	4	19.0	4	15.4	
No Recurrence	73	83.9	34	85.0	17	81.0	22	84.6	
Mortality in 30 days									
Death	3	3.4	2	5.0	0	0.0	1	3.8	0.793^C^
Survival	84	96.6	38	95.0	21	100.0	25	96.2	
Overall mortality									
Death	28	32.2	12	30.0	8	38.1	8	30.8	0.800^A^
Survival	59	67.8	28	70.0	13	61.9	18	69.2	
Cause of Death									
Tumor progression	9	10.3	5	12.5	2	9.5	2	7.7	0.739^A^
Postoperative complications	3	3.4	2	5.0	0	0.0	1	3.8	
Comorbidities	15	17.2	5	12.5	5	23.8	5	19.2	
Survival	59	67.8	28	70.0	13	61.9	18	69.2	
No information	1	1.1	0	0.0	1	4.8	0	0	

*CHO*, Cantonal Hospital Olten;* LUH*, Lausanne University Hospital;* LUMC*, Leiden University Medical Center;* SD*, Standard deviation;* UICC*, Union for International Cancer Control;* H&E*, hematoxilin and eosin;* OSNA*, one-step nucleic acid amplification;* Tx*, no information regarding Tumour size;* Lx*, no information regarding lymphatic invasion;* Vx*, no information regarding vascular invasion

^A^Chi-square test

^B^No test

^C^Kruskal–Wallis test

### Follow-up and outcome

The mean follow-up time was 53.4 months (SD ± 24.9). A total of 14 patients (16.1%) developed disease recurrence, with a mean time to recurrence of 19.8 months (SD ± 12.3). Thirteen patients had distant metastases; one patient was diagnosed with a local recurrence. The overall mortality was 32.2% (28 patients) from which 10.3% (nine patients) died due to recurrent disease.

### Survival analysis

**Fig. 1:**
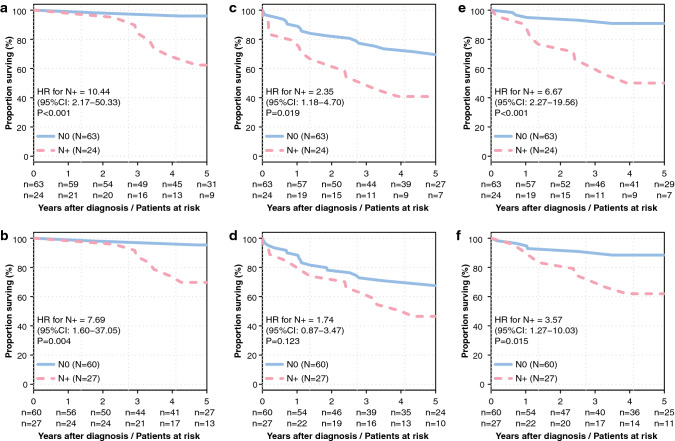
Univariate Kaplan–Meier survival curves according to diagnostic method; cancer-specific survival by H&E staining (**a**) and OSNA (**b**), disease-free survival by H&E staining (**c**) and OSNA (**d**), recurrence-free survival by H&E staining (**e**) and OSNA (**f**)

In univariable analysis, detection of positive LN with H&E staining was associated with worse DFS (HR = 2.35, 95% CI 1.18–4.69, *p* = 0.019), CSS (HR = 10.44, 95% CI 2.18–49.94, *p* < 0.001) and CSRFS (HR = 6.67, 95% CI 2.30–19.31, *p* < 0.001). For OSNA, no impact on DFS (HR = 1.74, 95% CI 0.88–3.43, *p* = 0.123), worse CSS (HR = 7.69, 95% CI 1.65–35.76, p *p* = 0.004) and CSRFS (HR = 3.57, 95% CI 1.29–9.85, *p* = 0.015) were observed (Fig. 1 and Table 2).

**Table 2:** Univariable and multivariable Cox regression analyses

	Cox regression for DFS				Cox regression for CSS				Cox regression for CSRFS			
	Univariable^A^		Multivariable^B^		Univariable^A^		Multivariable^B^		Univariable^A^		Multivariable^B^	
	HR (95% CI)	*p* ^C^	HR (95% CI)	*p* ^C^	HR (95% CI)	*p* ^C^	HR (95% CI)	*p* ^C^	HR (95% CI)	*p* ^C^	HR (95% CI)	*p* ^C^
OSNA												
Negative	Reference	0.123	Reference	0.873	Reference	0.004	Reference	0.197	Reference	0.015	Reference	0.727
Positive	1.74 (0.88–3.43)		0.93 (0.40–2.21)		7.69 (1.65–35.76)		3.08 (0.26–36.07)		3.57 (1.29–9.85)		1.25 (0.30–5.15)	
H&E staining												
Negative	Reference	0.019	Reference	0.064	Reference	< 0.001	Reference	0.019	Reference	< 0.001	Reference	0.017
Positive	2.35 (1.18–4.69)		2.56 (0.96–6.80)		10.44 (2.18–49.94)		10.77 (1.07–108.07)		6.67 (2.30–19.31)		5.96 (1.20–29.63)	
Age												
< 70 years	Reference	0.010	Reference	0.064	Reference	0.013	Reference	0.334	Reference	0.100	Reference	0.751
70 + years	2.66 (1.18–6.02)		2.19 (0.83–5.82)		7.92 (1.03–60.89)		2.84 (0.34–23.90)		2.48 (0.78–7.89)		1.23 (0.32–4.75)	
Gender												
Female	Reference	0.477	Reference	0.891	Reference	0.185	Reference	0.407	Reference	0.157	Reference	0.587
Male	1.29 (0.64–2.56)		1.05 (0.51–2.19)		2.68 (0.58–12.30)		2.21 (0.72–6.76)		2.19 (0.72–6.69)		1.42 (0.50–4.07)	
Tumour localization												
Ascending and Transverse Colon	Reference	0.033	Reference	0.080	Reference	0.277	Reference	0.469	Reference	0.027	Reference	0.078
Descending	3.65 (1.54–8.65)		3.28 (1.25–8.60)		2.56 (0.26–25.54)		5.20 (0.51–52.74)		5.36 (1.29–22.32)		7.28 (1.42–37.26)	
Sigmoid	1.69 (0.78–3.66)		1.29 (0.51–3.26)		3.06 (0.76–12.35)		3.13 (0.50–19.61)		3.89 (1.17–12.95)		3.18 (0.70–14.36)	
*T*-stage												
T1 and T2	Reference	0.369	Reference	0.885	Reference	0.115	Reference	0.335	Reference	0.226	Reference	0.731
T3 and T4	1.41 (0.68–2.92)		1.06 (0.48–2.34)		4.08 (0.52–32.26)		2.73 (0.43–17.39)		2.08 (0.59–7.27)		1.27 (0.33–4.87)	
Histological grade												
G1 and G2	Reference	0.744	Reference	0.616	Reference	0.647	Reference	0.351	Reference	0.761	Reference	0.416
G3	0.86 (0.37–1.97)		0.77 (0.32–1.90)		1.46 (0.31–6.91)		2.99 (0.41–21.65)		1.22 (0.38–3.94)		1.91 (0.47–7.78)	
Adjuvant therapy												
No	Reference	0.315	Reference	0.795	Reference	0.583	Reference	0.013	Reference	0.059	Reference	0.715
Yes	1.56 (0.66–3.70)		0.88 (0.31–2.50)		0.58 (0.08–4.12)		0.09 (0.01–1.34)		3.07 (1.09–8.62)		0.79 (0.21–2.97)	

*DFS*, disease-free survival;* CSS*, cancer-specific survival;* CSRFS*, cancer-specific and recurrence-free survival;* HR*, Hazard ratio with 95% confidence intervals* CI*, Wald type;* OSNA*, one-step nucleic acid amplification;* H&E*, haematoxylin and eosine

^A^Univariate cox regression analysis

^B^Multivariable cox regression analysis

^C^Likelihood ratio tests

Multivariate analysis confirmed detection of positive LN by H&E staining as a significant predictor for worse CSS (HR = 10.77, 95% CI 1.07–108.07, *p * = 0.019) and CSRFS (HR = 5.96, 95% CI 1.20–29.63, *p * = 0.017). For DFS (HR = 2.56, 95% CI 0.96–6.80, *p *  = 0.064), a tendency for worse survival was observed. In contrast, no impact of OSNA positivity on DFS (HR = 0.93, 95% CI 0.40–2.21,*p *= 0.873) CSS (HR = 3.08, 95% CI 0.26–36.07, *p * = 0.197) and CSRFS (HR = 1.25, 95% CI 0.30–5.15, *p *  = 0.727) persisted after multivariable adjustment (Table 2). The backward variable selection procedures uniformly confirmed the detection of positive LN by H&E but not by OSNA as significant predictors for CSS, DFS, and CSRFS.

### Diagnostic accuracy of H&E and OSNA

Considering cancer-specific death or recurrence as the gold standard for true LN positivity, sensitivity was 46.7% (95% CI 21.3–73.4%) for H&E and 60.0% (95% CI 32.3–83.7%) for OSNA. Specificity was 84.7% (95% CI 74.3–92.1%) and 75.0% (95% CI 63.4–84.5%) for H&E and OSNA respectively. The diagnostic odds ratio of H&E was 485.2 (95% CI 146.1–1611.8) compared to 450 (95% CI 140.7–1439.2) for OSNA.

Limiting the analysis to patients with a minimum of 12 harvested LN, sensitivity of H&E and OSNA were identical (66.7%; 95% CI 22.3–95.7%), while specificity of H&E was 75% (95% CI 47.9–92.7) and of OSNA 68.8% (95% CI 41.3–86.1).

### Time-dependent diagnostic accuracy of H&E and OSNA for survival prediction

**Fig. 2:**
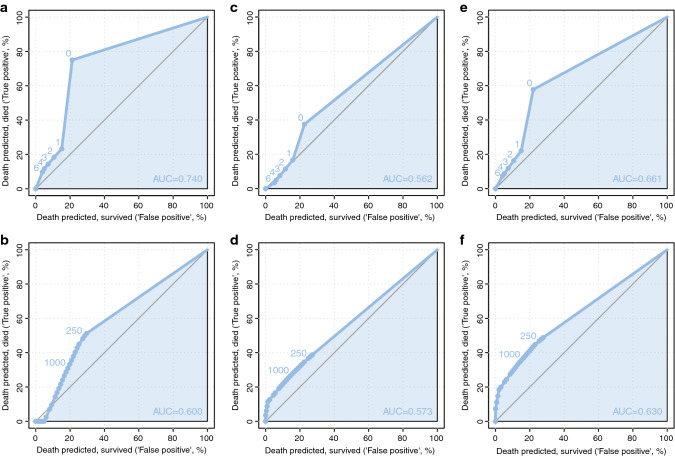
Time-dependent receiver operating characteristic (ROC) curves for 5-year survival rates. Curves for prediction by H&E staining are depicted in panels **a**, **c**, **e** and curves for prediction by OSNA in panel **b**, **d**, **f**. Panels **a** and **b** depict the disease-free survival, panels **c** and **d** depict the cancer-specific survival, and panels **e** and **f** depict the cancer-specific and recurrence-free survival. The area under the curve (AUC) is a measure for the diagnostic accuracy of the predictions with values of 1 representing a perfect prediction and values of 0.5 presenting a prediction by random. On the x-axis the false positives (death predicted, survived) and on the y-axis the true positives (death predicted, died) are shown. The true and false positives are estimated for each possible cut-off value (number of positive lymph nodes for H&E and Units for OSNA)

Both H&E and OSNA had a low to moderate predictive value for DFS (AUC of 0.562 and 0.573), CSS (AUC of 0.740 and 0.600) and CSRFS (AUC of 0.661 and 0.630) (Fig. 2). According to the ROC curves in Fig. 2 the cut-off for H&E staining, set to a minimum of one positive LN, obtains the best diagnostic value. For OSNA, the ROC curves suggest the possibility of higher AUC with cut-off values lower than 250 mRNA copies/μL, which were not assessed in the present study (Table 3). The diagnostic value of H&E was higher compared to OSNA for CSS and CSRFS: the observed sensitivity (defined as the rate of death predicted and not survived) and specificity (defined as 1 minus the rate of patients with death predicted and survived) for H&E staining for 5-year DFS were 37.6% and 77.0%, for 5-year CSS 75.0% and 78.8%, and for 5-year CSRFS 57.7% and 78.0%. The observed sensitivity and specificity for OSNA for 5-year DFS were 38.8% and 72.8%, for 5-year CSS 51.2% and 70.4%, and for 5-year CSRFS 48.8% and 72.2%.

**Table 3:** Lymph node positivity for different OSNA thresholds

OSNA threshold (copies/μL)	H&E	OSNA negative	OSNA positive
250	Negative	580	21
250	Positive	10	39
1000	Negative	593	8
1000	Positive	11	38
10,000	Negative	598	3
10,000	Positive	18	31

*OSNA*, one-step nucleic acid amplification;* H&E*, haematoxylin and eosin

## Discussion

This is the first study to evaluate the prognostic value of OSNA analysis in patients with colon cancer. According to the here presented results, OSNA offers no prognostic advantage compared to conventional LN staging with H&E. Interestingly, our previous studies identified OSNA as a promising diagnostic tool in colon cancer, showing high concordance rates with conventional histopathological workup with H&E, which were also confirmed by other authors in subsequent studies [13–15, 21]. However, all these previous trials investigated the performance of OSNA for the histopathological staging of colon cancer LN compared to either staging with H&E or multilevel sectioning and staining with immunohistochemistry and did not elucidate its prognostic value. Assessing the prognostic value of a method for LN staging is challenging as a reasonable gold standard for the definition of LN negativity, and positivity still does not exist. As a worldwide standard, one to two sections of each retrieved LN are analysed with H&E. Yet this workup entails a big risk of missing small nodal tumour infiltrates (i.e. isolated tumour cells and micrometastases), and this method is therefore not suitable as a gold standard against which a new diagnostic method can be tested for comparison. To compare OSNA with standard H&E workup, we therefore defined a gold standard for the definition of LN negativity and positivity. Assuming correctly staged node negative patients will not develop disease recurrence, we set the event of disease recurrence or cancer-related death as the gold standard to distinguish between true node negative and positive disease. With this distinct definition of true LN negativity and positivity, the diagnostic accuracy of H&E as well as of OSNA was markedly impaired compared to our previous reports as well as to a recently published systematic review [22]. This phenomenon occurs most likely due to missed small nodal tumour infiltrates at initial staging, suggesting that understaging is a persistent problem regardless of the technique used. The understaging is a relevant problem in the histopathological assessment of colon cancer, which was mentioned by our group previously, showing that multilevel sectioning with immunohistochemistry leads to a relevant upstaging of 15.4–26% of otherwise negatively classified patients [8, 23]. However, the prognostic impact of small nodal tumour infiltrates is still a matter of debate.

The previous studies focused on a dichotomic scale for LN positivity defining the threshold at 250 CK19 mRNA copies per µL, which corresponds to micrometastases, making sure that macro- and micrometastases and not isolated tumour cells are scored as metastatic LN involvement [10, 12–15, 17]. Since the present study is a retrospective analysis, the same threshold was considered.

The time-dependent ROC curves suggested the possibility of higher AUC with cut-off values lower than 250 mRNA copies/μL. This could be explained by the presence of isolated tumour cells. However, the previous studies did not characterize the CK 19 mRNA threshold corresponding to isolated tumour cells.

The amount of CK19 mRNA correlates with the size of the LN metastases making it feasible to calculate the total tumor load (TTL) of a specimen [21, 24]. These studies showed that the TTL correlates with the number of metastatic LN.

However, Aldecoa et al. observed that high-grade (G3) tumours or tumours with vascular invasion (V1) presented lower levels of TTL making it not a reliable prognostic tool for these specific pathologic features [24].

The multivariate cox analysis confirmed that a positive LN status with H&E (UICC stage III) was a predictor of worse CSS, as previously described in the literature [25, 26]. On the other hand, detection of positive LN with OSNA did not demonstrate to predict a worse CSS. This lack of significance may be due to the fact that fewer LN per patient were analysed with OSNA as only a part of a sentinel LN was used for OSNA analysis. In the case of H&E, all LNs were always examined by means of H&E, which is why there might be a higher chance of predicting recurrences. However, non-sentinel LN was only assessed with one to two H&E-stained sections, according to the worldwide standard. It would be necessary to fully analyse all LN of each patient by means of H&E and OSNA. This is technically not possible, as LN needed for H&E cannot be processed for OSNA and vice versa. However, by investigating the sentinel LN we set our focus on those LN having the highest probability of harbouring metastases. Nevertheless, we cannot ensure both LN fragments to harbour a metastasis, meaning that the metastasis could be found only in the fragment assigned to H&E analysis and not in the OSNA fragment and vice-versa.

The Kaplan–Meier survival analysis showed that a positive LN status with conventional H&E was predictive for worse CSS, DFS and CSRFS, in contrast to an OSNA-positive LN status.

We would like to acknowledge the limitations of the present study. Our sample size is relatively small which may result in reduced statistical power. Nevertheless, for the time being it is the largest published cohort of patients in the literature and the first study investigating the prognostic impact of OSNA in patients with colon cancer so far and includes patients from three different centres. As a cohort study, the presence of confounders is a possibility but the patients' characteristics of all considered study centres were homogenous, making a selection bias unlikely.

The present study is the first evaluating the prognostic value of OSNA in patients with colon cancer so far and includes patients from three different centres. OSNA failed to outperform conventional H&E workup regarding its prognostic value. Despite its relevance in other fields (breast cancer), the results of the here presented study do not support the adoption of OSNA alone in the routine staging of patients with colon cancer. Further studies should assess lower thresholds for OSNA (< 250 copies/μL), in order to detect isolated tumour cells increasing the prognostic value of this molecular detection method.
